# Double heterozygous pathogenic mutations in *KIF3C* and *ZNF513* cause hereditary gingival fibromatosis

**DOI:** 10.1038/s41368-023-00244-1

**Published:** 2023-09-26

**Authors:** Jianfan Chen, Xueqing Xu, Song Chen, Ting Lu, Yingchun Zheng, Zhongzhi Gan, Zongrui Shen, Shunfei Ma, Duocai Wang, Leyi Su, Fei He, Xuan Shang, Huiyong Xu, Dong Chen, Leitao Zhang, Fu Xiong

**Affiliations:** 1 Department of Medical Genetics, Experimental Education/Administration Center, School of Basic Medical Sciences, Southern Medical University, Guangzhou, China; 2 Experimental Department of Obstetrics and Gynecology Institute, The Third Affiliated Hospital of Guangzhou Medical University, Guangzhou, China; 3 Department of Precision Medicine, Shenzhen Hospital, Southern Medical University, Shenzhen, China; 4 Department of Stomatology, Nanfang Hospital, Southern Medical University, Guangzhou, China; 5 Department of Stomatology, The First Affiliated Hospital of Zhengzhou University, Zhengzhou, China; 6 Guangdong Provincial Key Laboratory of Single Cell Technology and Application, Guangzhou, China; 7 Department of Fetal Medicine and Prenatal Diagnosis, Zhujiang Hospital, Southern Medical University, Guangzhou, China

**Keywords:** CRISPR-Cas9 genome editing, Disease genetics

## Abstract

Hereditary gingival fibromatosis (HGF) is a rare inherited condition with fibromatoid hyperplasia of the gingival tissue that exhibits great genetic heterogeneity. Five distinct loci related to non-syndromic HGF have been identified; however, only two disease-causing genes, *SOS1* and *REST*, inducing HGF have been identified at two loci, GINGF1 and GINGF5, respectively. Here, based on a family pedigree with 26 members, including nine patients with HGF, we identified double heterozygous pathogenic mutations in the *ZNF513* (c.C748T, p.R250W) and *KIF3C* (c.G1229A, p.R410H) genes within the GINGF3 locus related to HGF. Functional studies demonstrated that the *ZNF513* p.R250W and *KIF3C* p.R410H variants significantly increased the expression of *ZNF513* and *KIF3C* in vitro and in vivo. *ZNF513*, a transcription factor, binds to *KIF3C* exon 1 and participates in the positive regulation of *KIF3C* expression in gingival fibroblasts. Furthermore, a knock-in mouse model confirmed that heterozygous or homozygous mutations within *Zfp513* (p.R250W) or *Kif3c* (p.R412H) alone do not led to clear phenotypes with gingival fibromatosis, whereas the double mutations led to gingival hyperplasia phenotypes. In addition, we found that ZNF513 binds to the *SOS1* promoter and plays an important positive role in regulating the expression of *SOS1*. Moreover, the *KIF3C* p.R410H mutation could activate the PI3K and KCNQ1 potassium channels. ZNF513 combined with KIF3C regulates gingival fibroblast proliferation, migration, and fibrosis response via the PI3K/AKT/mTOR and Ras/Raf/MEK/ERK pathways. In summary, these results demonstrate *ZNF513* + *KIF3C* as an important genetic combination in HGF manifestation and suggest that *ZNF513* mutation may be a major risk factor for HGF.

## Introduction

Hereditary gingival fibromatosis (HGF) is an inherited disease characterized by fibrous overgrowth with pathological, nonhemorrhagic, diffuse or focal, slowly progressive hyperplasia of the gingival tissue, resulting in fibromatous hyperplasia of the gingival tissue.^1,2^ HGF was first reported by Goddard and Gross in 1856 and has a prevalence of one in 175,000 individuals.^3,4^ Persistent gingival hyperplasia can lead to crowded dentition, inter-root interval, tooth displacement, and tooth eruption disorder, affecting the patient’s speech, difficulty in chewing, and aesthetics.^1,2^ Presently, gingival hyperplasia can only be treated by surgery. Since the disease is prone to recurrence after surgery, HGF patients must undergo repeated surgical procedures of gingival resection, which brings a heavy psychological and economic burden to HGF patients and their families.^5,6^ Therefore, investigation of the genetic etiology and molecular pathogenesis of HGF is important for its prevention, diagnosis, and treatment.

Most HGF cases are nonsyndromic, with gingival hyperplasia as the single symptom. In recent years, studies have shown that HGF can also be accompanied by other diseases such as hirsutism, epilepsy, mental retardation, hearing impairment, polydactyly, and growth hormone deficiency, which present as syndromic HGF, such as Ramon syndrome (OMIM 266270), Jones syndrome (OMIM 135550), and Hyaline fibromatosis syndrome (OMIM 228600).^1,7,8^ HGF is predominantly inherited in an autosomal dominant manner, with occasional cases of recessive inheritance and sporadic case reports.^9,10^ In addition, two Chinese pedigrees have been reported with maternal inheritance,^11^ suggesting that genomic imprinting may be associated with the pathogenesis of gingival fibroids. HGF presents with genetic heterogeneity, and nonsyndromic HGF is divided into five subtypes based on different candidate susceptibility genes or chromosomal loci, namely HGF types I–V, including GINGF1 on 2p22.1 (OMIM 135300),^12,13^ GINGF2 on 5q13-q22 (OMIM 605544),^14^ GINGF3 on 2p23.3-p22.3 (OMIM 609955),^15^ GINGF4 on 11p15 (OMIM 611010),^11^ and GINGF5 on 4q12 (OMIM 617626).^16^ Among these loci, “son of sevenless homologue 1” (*SOS1*, OMIM 182530) and “RE1 silencing transcription factor” (*REST*, OMIM 600571), which are HGF-related pathogenic genes, have been identified as associated with GINGF1 and GINGF5, respectively. *SOS1* was first identified in a Brazilian HGF family by Hart et al.^17^ *REST* was identified in three Turkish HGF families by Bayram et al.^16^ However, GINGF2, GINGF3, and GINGF4 were only found to be related to several non-syndromic HGF lineages, but none of the causative genes have been identified within these loci.^1,2^

In the present study, double heterozygous pathogenic mutations in the zinc finger protein 513 (*ZNF513*, OMIM 613598, NM_144631.6, c.C748T, p.R250W) and kinesin family member 3 C (*KIF3C*, OMIM 602845, NM_002254.8, c.G1229A, p.R410H) genes within the GINGF3 locus related to dominant HGF disease in a large family was identified and further confirmed by linkage analysis. Functional studies of these two mutations were performed using knock-in mice models with recapitulated fibrous overgrowth. Moreover, we further showed that ZNF513 interacts with KIF3C and affects *KIF3C* gene expression. In addition, mutations in the *ZNF513* and *KIF3C* genes increased the expression of the fibrosis markers *COL1A1* and *FN1* in vivo and in vitro. We conjectured that ZNF513, a transcription factor, combined with its target gene *KIF3C*, regulates the development of gingival tissue. Double mutations in *ZNF513* and *KIF3C* promote gingival fibroblast fibrosis, likely through the dysregulation of fibroblast proliferation, migration, and extracellular matrix synthesis, leading to HGF.

## Results

### Clinical evaluation of patients

**Fig. 1:**
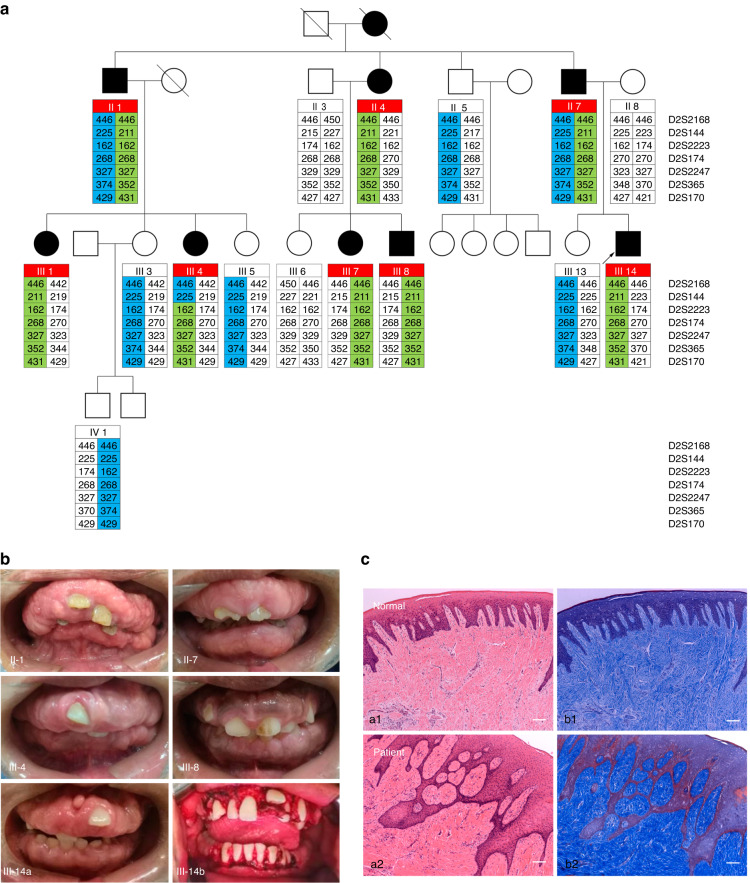
Pedigree, clinical features, and pathology of the affected individuals described in this study. **a** Pedigree and linkage study of 16 individuals selected from 23 subjects in the family. Arrow = proband; filled square = patient; green region = disease-associated haplotype; blue or white region = normal haplotype. **b** Clinical features of affected individuals, including II-1, II-7, III-4, III-8, and III-14. The bottom two pictures show the oral manifestations of the proband before (III-14a) and after gingivectomy (III-14b). **c** Paraffin section staining of normal gingiva and the patient’s gingival tumor tissue. HE staining: a1, a2 (10×). Masson staining: b1, b2 (10×). Scale bars indicate 100 μm

We identified a large Chinese family with autosomal dominant non-syndromic HGF according to intraoral diagnostic features. The family had nine affected members in a four-generation pedigree (Fig. 1a), and all family members were in otherwise good general health and had no abnormalities upon extraoral examination. Photographs and medical records permitted a diagnosis on the deceased individual I-2. The affected individuals presented severe gingival overgrowth involving the maxillary and mandibular arches, and had enlarged gingiva almost over the entire mandible and maxilla (Fig. 1b). No affected members presented with hearing loss, epilepsy, or hypertrichosis. HE and Masson staining of the gingiva from the proband showed that the upper and lower gingival masses were covered with stratified squamous epithelium and were associated with epithelial hyperplasia and subepithelial fibrous tissue hyperplasia with collagenization and were infiltrated with a large number of lymphocytes and plasma cells (Fig. 1c). According to the clinical and pathologic appearance, the patients were diagnosed with isolated, non-syndromic HGF.

### Genetic analysis

**Fig. 2:**
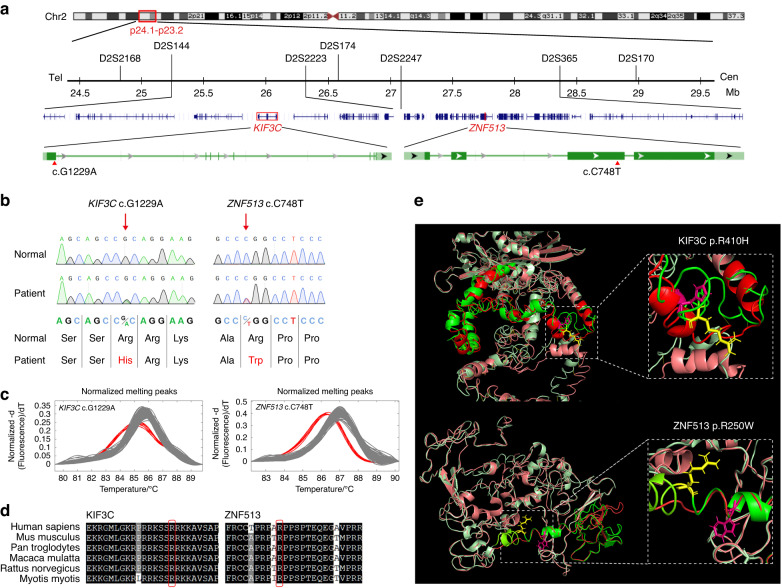
Mapping and identification of *KIF3C* and *ZNF513* mutations. **a** The segregating haplotype associated with HGF was inferred (boxed) with seven short tandem repeat markers on 2p24.1–p23.2. *KIF3C* was flanked by D2S144 and D2S2223, and *ZNF513* was flanked by D2S2247 and D2S365. The positions of the identified two mutations in *KIF3C* and *ZNF513* are indicated by red triangles. **b** Chromatograms of the genomic sequence of the two mutations. The top and bottom diagrams show differential sequences and proteins, respectively, and the arrow indicates the mutated basic group. **c** HRM analysis curves of the patients’ and 500 healthy volunteers’ gDNA at position *KIF3C* c.1229 and *ZNF513* c.748. Gray curves = control samples; red curves = patients. **d** Amino acid sequence alignment of the KIF3C and ZNF513 orthologues across different species. The arginine (R) residue at position 410 in KIF3C and residue at position 250 in ZNF513 are highly conserved. **e** Prediction of wild-type and mutant protein structures by I-TASSER and alignment by pymol. Mutant KIF3C and ZNF513 had clear differences in tertiary structure. Green = wild-type protein; red = mutant protein; yellow = wild-type amino acid; rose = mutant amino acid

In previous examinations, chromosome aberration in the proband was ruled out by array-CGH. Candidate genes from whole-exome sequencing were screened according to the American College of Medical Genetics and Genomics (ACMG) genetic variation classification criteria, pedigree separation, single nucleotide variant, or insertion-deletion variation in the exon regions, with pathogenicity prediction and phenotype-based prioritization (Supplementary Fig. 1). Double heterozygous variants of c.C748T (p.R250W) in *ZNF513* and c.G1229A (p.R410H) in *KIF3C* were validated by Sanger sequencing followed by co-segregation analysis in all affected and unaffected family members (Fig. 2b). In addition, we ruled out the previously reported four candidate regions for HGF by haplotype linkage analysis (data not shown), and there were no positive results for any of the known mutations in HGF-related genes, including duplication/deletion of *SOS1* and *REST*, based on the whole-exome data. *ZNF513* and *KIF3C* are located on chromosome 2p23.3, and this candidate region was confirmed by seven short tandem repeat markers on 2p (Figs. 1a, 2a). Furthermore, these two nucleotide changes were not detected in DNA samples from 500 normal unrelated controls matched for Chinese ethnicity (Fig. 2c). The Arg250 residue of ZNF513 and Arg410 residue of KIF3C are highly conserved across species from *Homo sapiens* to *Myotis myotis* (Fig. 2d). By aligning the protein structures predicted by ITASSER, we found that the amino acid changes in KIF3C and ZNF513 led to significant changes in both the secondary and tertiary structures comparing the peptide chains with and without these mutations (Fig. 2e).

### Expression and functional analysis of mutant *ZNF513* and *KIF3C*

To understand the effects of the *ZNF513* p.R250W and *KIF3C* p.R410H variants on the expression and function of tissues and cells, we analyzed them in biopsied gingival tissues, primary gingival fibroblasts, stable overexpressed HGF cell lines, and transient transfected NHGFs.

Expression and functional analysis of *KIF3C* and *ZNF513* in biopsied gingival tissues and primary gingival fibroblasts

**Fig. 3:**
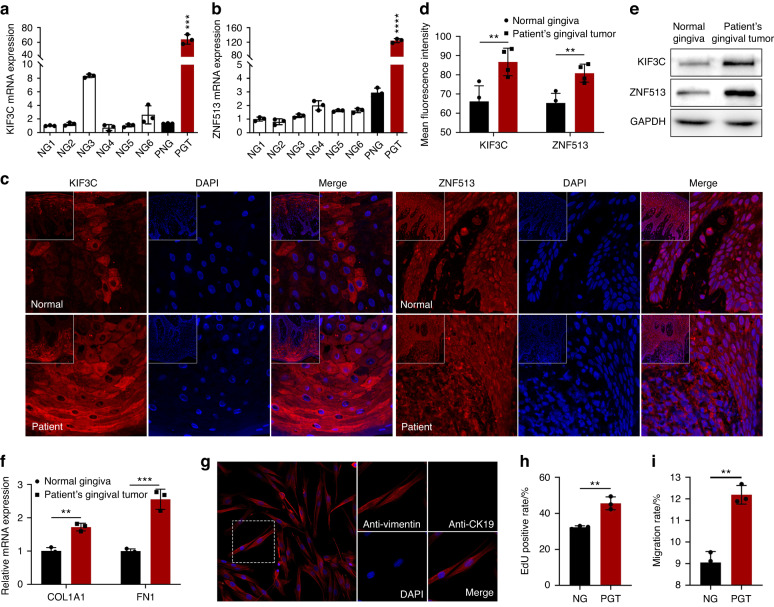
Effects of the two mutations on *KIF3C* or *ZNF513* expression and function in human gingival biopsies and primary gingival fibroblasts. **a**, **b** The mRNA expression levels of *KIF3C* and *ZNF513* in normal gingiva, and two mutants in the patient’s normal gingiva and the gingival tumor collected from six healthy random volunteers and the proband in the family, respectively. NG = normal gingiva; PNG = patient’s normal gingiva; PGT = patient’s gingival tumor. **c**, **d** By immunohistofluorescence analysis, the mean fluorescence intensity of KIF3C and ZNF513 in the patient’s gingival tumor were significantly increased compared to normal gingiva. Confocal images of KIF3C and ZNF513 (red), DAPI nuclear staining (blue), and merged signals. Object lens up to 63 magnifications. **e** By immunoblot analysis, the protein levels of KIF3C and ZNF513 in the patient’s gingival tumor were significantly increased. **f** The expression levels of fibrosis markers, *COL1A1* and *FN1*, in the patient’s gingival tumor were significantly increased. **g** By immunofluorescence analysis, primary human gingival fibroblasts were isolated successfully and were identified as being anti-Vimentin positive (red) and anti-CK19 negative. **h**, **i** Compared with normal primary gingival fibroblasts, the proliferation (**h**) and migration (**i**) of the patient’s gingival tumor fibroblasts were increased. *P < 0.05; **P < 0.01; ***P < 0.001; ****P < 0.000 1

The expression of *KIF3C* and *ZNF513* in patient’s normal gingiva tissue was not significantly different from that in the control group, but the expression in tumor tissue was significantly increased (Fig. 3a, b, e; Supplementary Fig. 2a). Simultaneously, immunohistofluorescence detection showed that two proteins had stronger fluorescence intensity in gingival tumors (Fig. 3c, d). In addition, we found a significant increase in the expression of the fibrosis markers collagen type I alpha 1 chain (*COL1A1*, OMIM 120150) and fibronectin 1 (*FN1*, OMIM 135600) in the tumor tissues of the proband (Fig. 3f). Excessive production of extracellular matrix (ECM), driven by COL1A1, may be the cause of overgrowth of gingival fibroblasts.^18,19^ Glycoprotein fibronectin, another important component of the ECM, is secreted in the form of a dimer protein and assembled into fibers on the cell surface.^20^

Fibroblasts were isolated from fresh human gingival tissues, and primary culture was performed up to the third or fourth generation of cells, which were used for follow-up experiments. The isolated primary cells expressed vimentin specific to fibroblasts, while epithelial cytokeratin staining was negative (Fig. 3g). In the patient’s primary gingival tumor fibroblasts, we found that the increased expression of *KIF3C* or *ZNF513* was consistent with the *KIF3C* or *ZNF513* expression in patient’s gingival tumor tissues (Supplementary Fig. 2b), and tumor fibroblasts had a stronger ability to proliferate and migrate after 24 h cultured compared with NHGFs (Fig. 3h, i; Supplementary Fig. 3a, d; Supplementary Fig. 4b).

Expression and functional analysis of *KIF3C* and *ZNF513* in stable lentivirus overexpressed HGF cell lines and transiently transfected NHGFs

**Fig. 4:**
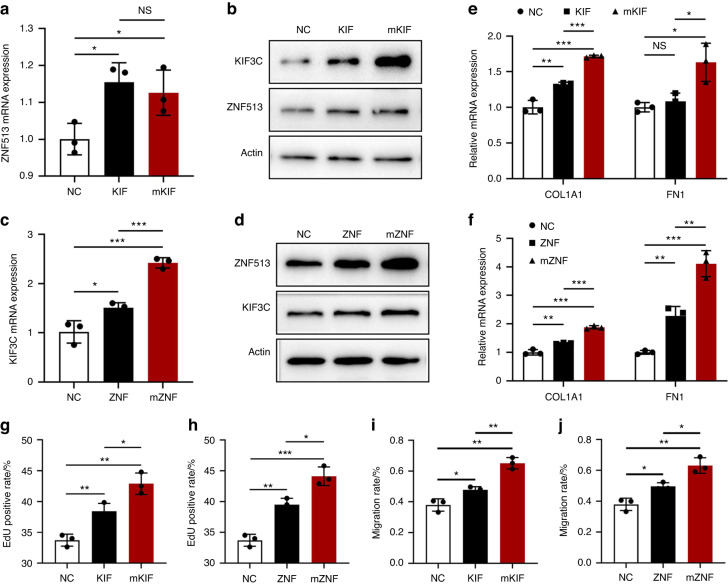
Effects of the two mutations on the expression and function in stable lentivirus overexpressing *KIF3C* and *ZNF513* cell lines. **a**, **b** The mRNA expression levels and the protein levels of the two genes in HGF-KIF3C and HGF-mKIF3C cell lines. **c**, **d** The expression levels of the two genes in HGF-ZNF513 and HGF-mZNF513 cell lines. **e**, **f** The expression levels of *COL1A1* and *FN1* in HGF-mKIF3C and HGF-mZNF513 cell lines were significantly increased. **g**–**j** Effects of two missense mutants on proliferation (**g** and **h**) and migration (**i** and **j**) in four overexpression cell lines. NC = HGF-pLenti cell line; (m)KIF = HGF-(m)KIF3C cell line; (m)ZNF = HGF-(m)ZNF513 cell line; NS = not significant

To further understand the effects of *ZNF513* and *KIF3C* in NHGFs, we packaged lentiviruses containing empty pLenti-Bi-cistronic plasmids, overexpressed wild-type ZNF513-pLenti, KIF3C-pLenti plasmids, and mutant plasmids, and then successfully infected NHGFs. After puromycin screening and expanded culture, we obtained the following five cell lines: HGF-pLenti, HGF-KIF3C, HGF-mKIF3C, HGF-ZNF513, and HGF-mZNF513. In overexpressed cell lines, the expression of *ZNF513* and *KIF3C* were significantly increased at the RNA level, and the expression in mutant cell lines was higher. The mRNA expression of *KIF3C* was significantly increased in HGF-mZNF513, but the expression of *ZNF513* was not affected in HGF-mKIF3C (Fig. 4a, c; Supplementary Fig. 2c, e). There was also a similar trend at the protein level (Fig. 4b, d; Supplementary Fig. 2d, f), suggesting that ZNF513 may play a promoting role in the expression of *KIF3C*. In addition, the expression of *COL1A1* and *FN1* increased to varying degrees (Fig. 4e, f), proliferation and migration increased (Fig. 4g–j; Supplementary Fig. 3b, e; Supplementary Fig. 4c), and the effect of mutant cell lines were more apparent after overexpression. These observations suggest that excessive expression of *ZNF513* and *KIF3C* may be associated with cell fibrosis, proliferation, and migration.

Moreover, we constructed pcDNA3.1flag-(m)KIF3C and pEGFPN1-(m)ZNF513 overexpression plasmids to transiently transfect NHGFs and found that the expression levels were similar to those of lentivirus overexpression cell lines (Supplementary Fig. 5a, b). CCK-8 detection also showed that cells proliferated faster with mutant ZNF513 and KIF3C after transient transfection (Supplementary Fig. 5d). Immunofluorescence showed that the cellular localization of these proteins did not change in the presence of the mutations (Supplementary Fig. 5c).

### ZNF513 binds to *KIF3C* exon 1 and participates in the regulation of *KIF3C* in gingival fibroblasts

**Fig. 5:**
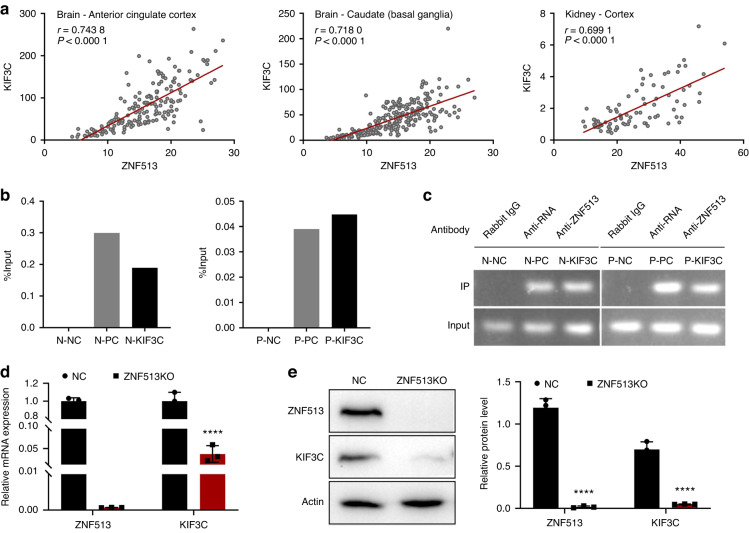
Confirmation of the binding and regulation of ZNF513 to *KIF3C*. **a** The expression of *KIF3C* and *ZNF513* derived from the GTEx database in 54 tissues showed that they are co-expressed and positively correlated in the brain, kidney, and other tissues. Each grey dot represents one sample. Red lines are regression equations. **b**, **c** ChIP was performed in NHGFs and patient’s gingival tumor fibroblasts by ZNF513 antibody and control antibodies, respectively. ChIP-qPCR and agarose gel electrophoresis showed that ZNF513 binds to *KIF3C* exon 1. N-NC, N-PC, and N-KIF3C indicate negative control, positive control, and experiment group (ZNF513 antibody), respectively, in NHGFs. P-NC, P-PC, and P-KIF3C indicate negative control, positive control, and experiment groups of the patient’s gingival tumor fibroblasts, respectively. **d**, **e** A *ZNF513* knockout HGF cell line was constructed with CRISPR/Cas9, and the mRNA and protein levels of KIF3C showed a significant decrease. NC = HGF-lentiCRISPR

A previous study found that ZNF513 is a transcription factor that binds and regulates the expression of other genes.^21^ Based on our results, increased *KIF3C* expression was detected in the HGF-ZNF513 cell line and NHGFs with transient overexpression of wild-type *ZNF513* plasmids, so we speculated that ZNF513 is a positive regulator of *KIF3C*. The expression of *ZNF513* and *KIF3C* were extracted from 54 different human tissues from the GTEx database. We found that they are co-expressed and positively correlated in many tissues, and negative correlation was found in a few tissues (Fig. 5a). Table S1 lists 12 different human tissues’ information that meet the filtering criteria | r | > 0.6 and *p* < 0.001. The Cistrome DB database contains ChIP-seq data on ZNF513 in HEK293 cells, which shows that ZNF513 binds to *KIF3C* and has an obvious enrichment peak in exon 1 of *KIF3C* (Supplementary Fig. 6e). Therefore, we further studied the binding and regulation of ZNF513 and KIF3C in gingival fibroblasts.

ChIP was performed in NHGFs and in patient gingival tumor fibroblasts. The results of ChIP-qPCR and electrophoresis confirmed that ZNF513 in two kinds of cells clearly showed an *KIF3C* exon 1 binding enrichment peak (Fig. 5b, c). The binding sequence was sequenced using T-A clones and was confirmed to be the genomic sequence of *KIF3C* (Supplementary Fig. 6f). Furthermore, CRISPR/Cas9 gene editing was used to carry out *ZNF513* knockout experiments in NHGFs, and the knockout effect of monoclones was verified by sequencing (Supplementary Fig. 6a). In the *ZNF513* knockout HGF cell line, the expression of *KIF3C* decreased significantly, showing almost no expression at the RNA and protein levels (Fig. 5d, e), suggesting that ZNF513 plays an important positive regulatory role on the expression of *KIF3C*.

### Phenotypic and functional studies of C57BL/6-*Kif3c*^R412H^ and C57BL/6-*Zfp513*^R250W^ knock-in mouse models

**Fig. 6:**
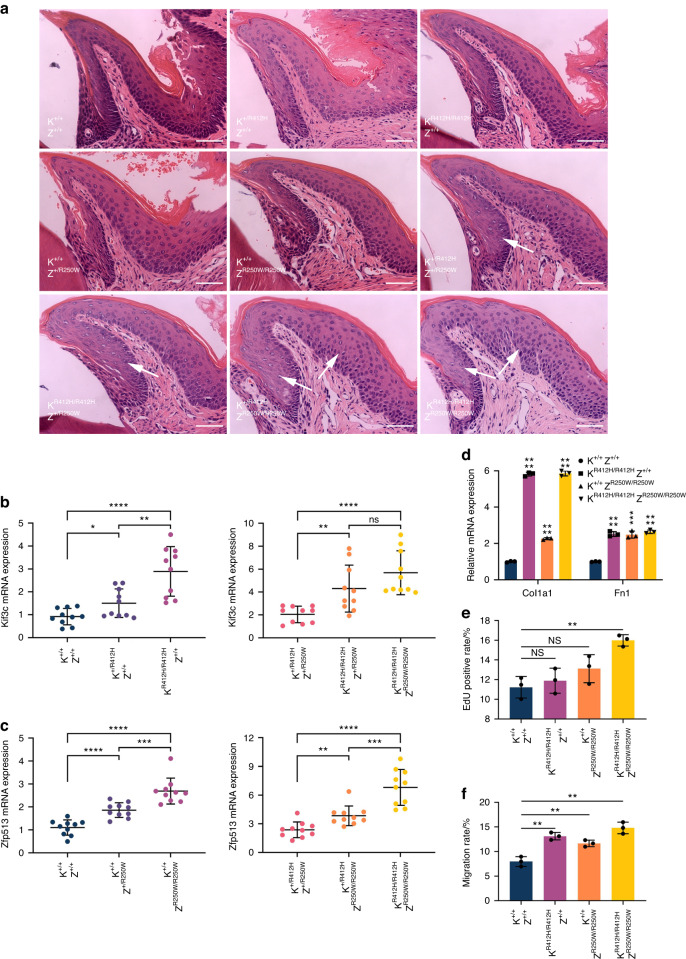
Differences in the phenotype and function of C57BL/6-*Kif3c*^R412H^ and C57BL/6-*Zfp513*^R250W^ knock-in mice. **a** HE staining of mandibular gingival tissue sections of 6-month-old male mice showed that the gingival epithelium of *Kif3c*^+/R412H^/*Zfp513*^+/R250W^, *Kif3c*^R412H/R412H^/*Zfp513*^+/R250W^, *Kif3c*^+/R412H^/*Zfp513*^R250W/R250W^, and *Kif3c*^R412H/R412H^/*Zfp513*^R250W/R250W^ genotypic mice thickened to a certain extent. Object lens up to 40 magnifications. Scale bars indicate 100 μm. **b**, **c** The expression of *Kif3c* and *Zfp513* in the maxillary gingiva of male mice increased in the mice with genetic mutations and was higher in those with the double mutation. Homozygous mutant mice expressed higher compared with heterozygous mice. **d** The expression levels of *Col1a1* and *Fn1* were significantly increased in the maxillary gingiva of male mice with the *Kif3c*^R412H/R412H^/*Zfp513*^+/+^, *Kif3c*^+/+^/*Zfp513*^R250W/R250W^, and *Kif3c*^R412H/R412H^/*Zfp513*^R250W/R250W^ genotypes. **e**, **f** The proliferation (**e**) and migration (**f**) of primary gingival fibroblasts from male mice were detected to show that the mice with the *Kif3c*^R412H/R412H^/*Zfp513*^R250W/R250W^ genotype had stronger proliferation and migration ability

Here, we established transgenic *Kif3c*^R412H^ and *Zfp513*^R250W^ knock-in mouse models. Human *KIF3C* and *ZNF513* are located on chromosome 2, while in mice, they are located on chromosomes 12 and 5, respectively. The homologue of the human *KIF3C* p.R410 in the mouse is at position p.R412. The normal birth frequency of knock-in mice followed the classical Mendelian genetic pattern. Four weeks after decalcification, the mandible of 6-month-old male mice was sectioned at the first molar and stained with HE. The results showed that the gingival epithelium of *Kif3c*^+/R412H^/*Zfp513*^+/R250W^, *Kif3c*^R412H/R412H^/*Zfp513*^+/R250W^, *Kif3c*^+/R412H^/*Zfp513*^R250W/R250W^, and *Kif3c*^R412H/R412H^/*Zfp513*^R250W/R250W^ mice thickened to some extent, and the gingival epithelium of the latter two genotypes increased significantly (Fig. 6a). The expression of *Kif3c* and *Zfp513* was increased in the maxillary gingiva of mutant knock-in mice, and was higher in mice with both mutations (Fig. 6b, c). We randomly took five samples from the maxillary gingival RNA of wild-type mice and from male mice with three gene mutations (*Kif3c*^R412H/R412H^/*Zfp513*^+/+^, *Kif3c*^+/+^/*Zfp513*^R250W/R250W^, and *Kif3c*^R412H/R412H^/*Zfp513*^R250W/R250W^) and mixed them in equal proportions. We found that the expression levels of the fibrosis markers *Col1a1* and *Fn1* in mutant mice were significantly increased (Fig. 6d). The maxillary gingival tissues of mice are smaller, so they are not conducive for culturing primary cells. Therefore, we also took five fresh maxillary gingival tissue from the wild-type mice and three male mice with genetic mutants for primary cell culture to the third generation for cell proliferation and migration detection. The results showed that the proliferation and migration ability of *Kif3c*^R412H/R412H^/*Zfp513*^R250W/R250W^ genotype mice were greater (Fig. 6e, f; Supplementary Figs. 3c, 4d).

### Exploration of the pathogenic mechanism of *ZNF513* and *KIF3C*

**Fig. 7:**
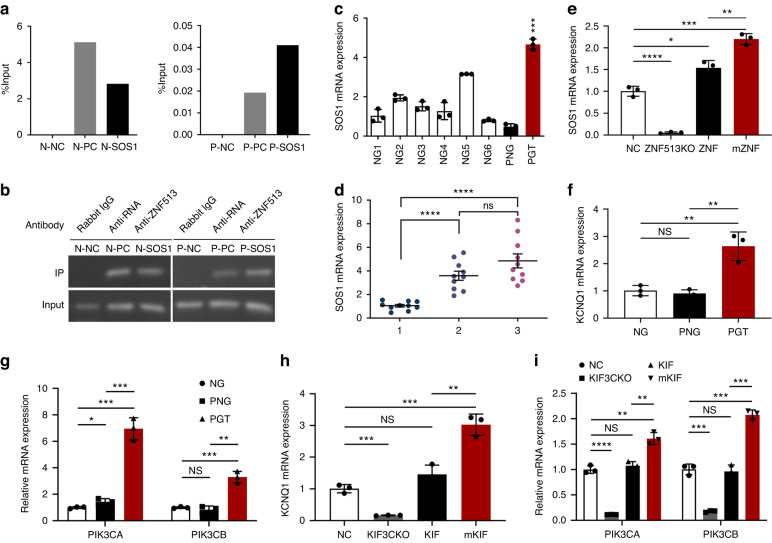
Exploring the pathogenic mechanism of KIF3C and ZNF513. **a**, **b** ChIP-qPCR and agarose electrophoresis of NHGFs and patient’s gingival tumor fibroblasts showed that ZNF513 binds to the promoter of *SOS1*. N-SOS1 and P-SOS1 indicate experiment groups in NHGFs and in patient’s gingival tumor fibroblasts, respectively. **c** Compared with normal gingiva and patient’s normal gingiva, the expression of *SOS1* in the patient’s gingival tumor tissue was significantly higher. **d** The expression of *Sos1* increased in the maxillary gingiva of 6-month-old male mice with *Zfp513* mutation. **e** The expression of *SOS1* in the *ZNF513* knockout HGF cell line decreased significantly, while in the HGF-mZNF513 cell line, the mRNA level increased significantly. **f**, **g** The mRNA expression of *KCNQ1*, *PIK3CA*, and *PIK3CB* in the gingival tumor tissue of the patient was significantly higher. **h**, **i** The expression of *KCNQ1*, *PIK3CA*, and *PIK3CB* decreased significantly in the *KIF3C* knockout HGF cell line but increased in the HGF-mKIF3C cell line. 1 = *Kif3c*^+/+^/*Zfp513*^+/+^ mice; 2 = *Kif3c*^+/+^/*Zfp513*^+/R250W^ mice; 3 = *Kif3c*^+/+^/*Zfp513*^R250W/R250W^ mice; NC = NHGFs

*SOS1*, located at 2p22.1, is a known pathogenic genes related to HGF. Based on the analysis of GTEx database, *SOS1* and *ZNF513* are co-expressed and positively correlated in many tissues. A Venn diagram was drawn to represent the intersection of the co-expressed tissues of *ZNF513* and *KIF3C* or *SOS1* with a filtering condition of | r | > 0.5 (Supplementary Fig. 6d). The ChIP-seq dataset of ZNF513 from the Cistrome DB database showed that ZNF513 binds to *SOS1* and has a clear enrichment peak in the promoter of *SOS1* in HEK293 cells (Supplementary Fig. 6e). The ChIP-qPCR and electrophoresis of NHGFs and patient gingival tumor fibroblasts confirmed that ZNF513 in these cells had clear binding at the enrichment peak of the *SOS1* promoter region (Fig. 7a, b), and the binding sequence was confirmed to be the genomic sequence of *SOS1* (Supplementary Fig. 6f). Compared with normal gingival tissue, the expression of *SOS1* in gingival tumors from the proband was abnormally increased, whereas no variants in *SOS1* gene were found in all patients of the family (Fig. 7c). In the maxillary gingiva of 6-month-old male mice, the expression of *Sos1* in *Zfp513* mutant mice was relatively high (Fig. 7d). The mRNA expression of *SOS1* was increased in the HGF-mZNF513 cell line but decreased significantly in the *ZNF513* knockout HGF cell line, and had almost no detectable expression (Fig. 7e). These results suggest that the transcription factor ZNF513 may play an important positive regulatory role in the expression of *SOS1*.

In gingival tissue biopsy, we found that the mRNA expression level of *KCNQ1*, *PIK3CA*, and *PIK3CB* in the gingival tumor tissue of the proband was significantly increased (Fig. 7f, g). KCNQ1, also known as Kv7.1, is a member of the voltage-gated potassium channel family.^22^ *PIK3CA* and *PIK3CB* encode p110 α and p110 β, respectively, which are the two catalytic subunits of the PI3K enzyme. To further confirm the pathogenic mechanism of KIF3C, we used CRISPR/Cas9 gene editing to carry out a *KIF3C* knockout experiment in NHGFs and verified the knockout effect of monoclones by sequencing and mRNA and protein expression level detection (Supplementary Fig. 6b, c). In vitro, the mRNA expression of *KCNQ1*, *PIK3CA*, and *PIK3CB* increased in the HGF-mKIF3C cell line but decreased significantly in the *KIF3C* knockout HGF cell line (Fig. 7h, i), suggesting that mutation of *KIF3C* may be related to the activation of the PI3K and KCNQ1 potassium channels.

## Discussion

In this study, we reported double heterozygous missense mutations in *ZNF513* and *KIF3C*, causing HGF in a large family including nine patients. Both *ZNF513* and *KIF3C* are located on chromosome 2p23.3, within the reported pathogenic region 2p23.3–p22.3 of HGF GINGF3. This candidate region was further confirmed by additional data from short tandem repeat markers on 2p in this large family. Furthermore, we constructed the compound mutation of *ZNF513* and *KIF3C* in a knock-in mouse model to recapitulate the features of the HGF. HGF is a group of genetic disorders with high clinical and genetic heterogeneity. Nonsyndromic HGF is divided into five subtypes based on different candidate susceptibility genes or chromosomal loci.^1^ However, apart from the reported *SOS1* and *REST* genes associated with GINGF1 and GINGF5,^16,17^ respectively, no pathogenic genes associated with the other three subtypes have been identified. Here, we found that double heterozygous mutations in *KIF3C* and *ZNF513* cause HGF associated with GINGF3, which adds new pathogenic genes for HGF and expands the pathogenic gene spectrum for HGF.

In our study, we found that *ZNF513* (c.C748T) and *KIF3C* (c.G1229A) mutations co-segregated with the HGF phenotype in family members, and neither of these two mutations were detected in 300 ancestry-matched unaffected individuals. Furthermore, we observed that *ZNF513* and *KIF3C* expression was significantly increased in patient gingival tumor tissue and primary gingival fibroblasts. Subsequently, we detected the high expression of *ZNF513* or *KIF3C* in stable HGF-mZNF513 or HGF-mKIF3C cell lines. *KIF3C* expression was increased in the HGF-mZNF513 cell line, whereas *ZNF513* expression was not changed in the HGF-mKIF3C cell line. Similar results were found in transiently transfected NHGF experiments. These results indicate that the p.R250W variant of ZNF513 positively affects the expression of *ZNF513* and *KIF3C*, whereas the p.R410H variant of *KIF3C* has no effect on the expression of other genes, such as *ZNF513*, except for the increase of its own expression. The binding of ZNF513 to KIF3C in human gingival fibroblasts was further confirmed by analysis of GTEx data, Cistrome DB, and the ChIP-qPCR experiment. In vitro, the expression of *KIF3C* increased in the HGF-ZNF513 cell line but decreased significantly in the *ZNF513* knockout HGF cell line, suggesting that ZNF513 plays an important positive regulatory role in the expression of *KIF3C*. In addition, the expression of the fibrosis markers *COL1A1* and *FN1* was increased in mutant overexpression HGF cell lines and knock-in mice with *Zfp513* and *Kif3c* mutations, indicating that both *ZNF513* and *KIF3C* mutations affect the formation of gingival cell fibrosis.

**Fig. 8:**
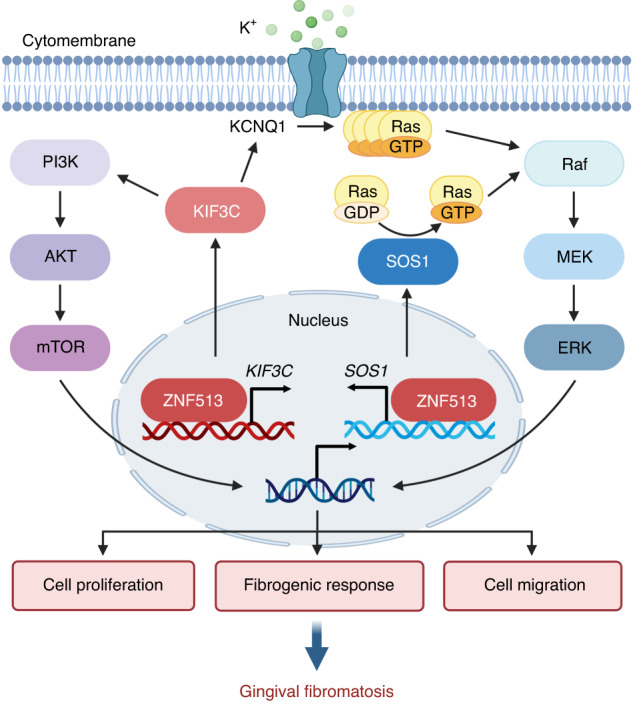
Mutant ZNF513 and KIF3C respond to signals inside and outside the nucleus via the PI3K/AKT/mTOR and Ras/Raf/MEK/ERK pathways to modulate the expression of fibrotic genes, improve cell proliferation and the migration and fibrogenic response, and eventually lead to the occurrence of gingival fibroma. Mutant KIF3C activates PI3K, and the activated PI3K transmits signal cascades to activate the downstream AKT and mTOR.^23,25^ Mutant KIF3C activates the KCNQ1 potassium channel and induces active Ras binding to GTP to activate on the cytomembrane. Activated Ras then promotes the activation of Raf and MEK and finally promotes the phosphorylation and nuclear translocation of ERK.^26^ ZNF513 binds to the *KIF3C* promoter and regulates its expression. The increased expression of *KIF3C* regulated by mutant ZNF513 further promotes the activation of PI3K and potassium channels. In addition, SOS1 catalyzes the release of GDP and accelerates the binding of GTP to Ras. Activated Ras feeds into the MAPK cascade by activating Raf recruited to the membrane.^24^ The combination of mutant ZNF513 with the *SOS1* promoter and the regulation of its increased expression promotes this process

On the other hand, we found that the *KIF3C* p.R410H mutation can enhance the expression of *PIK3CA* and *PIK3CB*. High expression of *PIK3CA* and *PIK3CB* leads to continuous activation of the PI3K enzyme. The PI3K/AKT/mTOR pathway is a key mechanism to control cell survival, division, and metabolism. Activated PI3K can phosphorylate PIP2, generate PIP3 on the cytomembrane, and transmit signals to activate downstream AKT and mTOR, thus causing cell proliferation and motility.^23,24^ Studies have shown that excessive expression of *KIF3C* can promote cell proliferation, migration, and invasion and inhibit cell apoptosis by activating the PI3K/AKT/mTOR pathway, leading to glioma.^25^ *KIF3C* p.R410H also contributes to the increased expression of *KCNQ1*. Previous research found that activation of the KCNQ1 potassium channel can affect the expression of fibrogenic genes and enhances the fibrotic activity of NHGFs via Ras/MAPK/AP-1 signaling.^26^ ZNF513 also binds to the *SOS1* promoter by ChIP-qPCR, and we found that *ZNF513* (*Zfp513*) p.R250W could increase the expression of *SOS1* (*Sos1*) in vivo, in vitro, and in mouse experiments, while the expression of *SOS1* significantly decrease in *ZNF513* knockout HGF cell line. SOS1 is a bifunctional guanosine monophosphate exchange factor that is down-regulated under normal physiologic condition, and its carboxyl terminal domain plays a negative regulatory role.^1,27^ The activation of SOS1 can catalyze Ras GTP exchange and indirectly increase the activity of the mitogen-activated protein kinase (MAPK) signaling pathway through Ras phosphorylation, which induces extracellular signal-regulated kinase (ERK) phosphorylation through cascade reactions. Ras activation is a key mechanism regulating cell survival, proliferation, and differentiation, as well as gene transcription.^24,28^ *SOS1* regulates fibroblast proliferation and migration through the Ras/ERK pathway.^24,29^ Therefore, activation of the KCNQ1 potassium channel and increased *SOS1* expression promote the fibrogenic response and proliferation and migration of gingival fibroblasts through the Ras/Raf/MEK/ERK pathway. We deduced that *ZNF513* mutation led to the elevated expression of *KIF3C* and *SOS1*; meanwhile, *KIF3C* mutation led to the activation of PI3K and elevated *KCNQ1* expression, which led to accelerated fibrosis of gingival fibroblasts and the enhanced proliferation and migration of fibroblasts, and finally led to the development of gingival fibromatosis (Fig. 8).

KIF3C is a molecular chaperone of KIF3A, and together they generate the heterodimer KIF3AC to participate in intracellular transport.^30^ *KIF3C* is highly expressed in glioma, small cell lung cancer, esophageal cancer, and other cancers.^31,32^ It is even considered as a potential therapeutic target for solid tumors; for example, reducing the expression of *KIF3C* inhibits the proliferation and metastasis of breast cancer.^31,33^ The above studies suggest that high expression of *KIF3C* may cause a disruption of cell division leading to tumorigenesis. The *KIF3C* c.G1229A mutation led to a significant increase in *KIF3C* expression at the mRNA and protein levels, and also significantly increased cell proliferation and migration, showing the efficacy of gain of function. However, no reports of diseases caused by *KIF3C* mutation including in gingival fibromatous disease have been published. Moreover, in our study, we found that heterozygous or homozygous mice with *Kif3c* mutation alone did not exhibit symptoms of gingival fibromatosis. Thus, we speculate that the high expression of this gene resulting from this mutation may affect the differentiation of gingival fibroblasts and promote the development of disease; however, mutation of *KIF3C* alone is not sufficient to cause the development of gingival fibromatosis.

ZNF513, a zinc finger protein, is widely expressed in various tissues of the body and plays an important role in gene regulation. However, there are few reports on the physiological functions of *ZNF513*. Li et al. reported that homozygous mutation of *ZNF513* (c.T1015C, p.C339R) can cause autosomal-recessive retinitis pigmentosa,^21^ but no retinopathy was found in the homozygous mutant mice with *Zfp513* p.R250W in our study, which shows that variants at different sites of *ZNF513* display clinical heterogeneity. In addition, Li et al. used ChIP analysis to reveal that ZNF513 binds to the *Arr3*, *Sp4*, *Pax6*, *Irbp*, and photoreceptor opsin promoters.^21^ In our study, we performed ChIP analysis of the genes that bind to ZNF513 in HEK293 cells as indicated in the Cistrome DB database, and the results showed that ZNF513 binds to *KIF3C* and *SOS1* in gingival fibroblasts. In stable cell lines with lentivirus overexpressing *ZNF513* and a *ZNF513* knockout HGF cell line, we found that ZNF513 played an important positive role in regulating the expression of *KIF3C* and *SOS1*. Moreover, the *ZNF513* c.C748T mutation enhanced the expression of *KIF3C* or *SOS1*, thus indirectly promoting the proliferation and migration of gingival fibroblasts and the development of gingival tumors.

To investigate the pathogenicity of the combinations of *ZNF513* and *KIF3C* variants, we introduced double mutants into mice. Knock-in mice carrying *Zfp513* + *Kif3c* double variants, especially in *Kif3c*^R412H/R412H^/*Zfp513*^R250W/R250W^ mice, had gingiva that were thickened to a certain extent. However, the gingival tissues were not thickened in mice with either *Kif3c* or *Zfp513* variant or in homozygous variants alone. The knock-in mice did not have glaringly obvious gingival thickening like that of patients. The reason may be that mice teeth grow continuously in a somewhat different manner from that of human teeth. The development of mice teeth including gingiva may be controlled by different factors. In addition, human *KIF3C* and *ZNF513* are located nearby to each other on chromosome 2; however, *Kif3c* in mice is located on chromosome 12, and *Zfp513* is located on mouse chromosome 5. This may result in biological effects of *Zfp513 + Kif3c* in mice that are not completely similar in humans. Moreover, the proliferation and migration of *Kif3c*^R412H/R412H^/*Zfp513*^R250W/R250W^ mice gingival fibroblasts were significantly enhanced, and the expression of fibrosis markers were clearly increased, indicating that double mutations of *Kif3c* combined with *Zfp513* could promote a gingival fibrogenic response and affect the proliferation, migration, and extracellular matrix synthesis of mice gingival fibroblasts.

In conclusion, we identified mutations in two pathogenic genes, *ZNF513* and *KIF3C*, which jointly cause HGF. A knock-in mouse model with compound mutations of *Zfp513* and *Kif3c* recapitulated the hallmark features of the disease. ZNF513 combined with KIF3C via the PI3K/AKT/mTOR and Ras/Raf/MEK/ERK pathways regulated the proliferation, migration, and fibrosis response of gingival fibroblasts. Our study demonstrates *ZNF513* + *KIF3C* as an important genetic combination in HGF manifestation, and suggests that ZNF513 may be a major risk factor for the development of HGF.

## Materials and methods

### Study participants and sample collection

This study, including the clinical examination and gingival tissue biopsies, was approved and performed in accordance with the ethical committee standards at the Southern Medical University, China. Patients and relatives analyzed in the project signed informed consent prior to inclusion. All subjects from the same family with non-syndromic HGF and six random subjects (control samples) were recruited from the Department of Stomatology at Nanfang Hospital. The patients’ medical records and medication histories were collected by a skilled clinician. In addition, eight unaffected individuals from the family underwent oral and clinical examinations.

Peripheral venous blood was collected from sixteen family members. Gingival biopsies from the proband with non-syndromic HGF was collected during gingivectomy, which was carried out to excise excessive abnormal proliferation of the coronal/marginal part of the gingiva. Control gingival biopsies were collected from six healthy random volunteers, aged 20–30 years, due to wisdom tooth extraction. After collection, the gingival tissues were immediately placed into DMEM/F12 (10% FBS) and rinsed with sterile phosphate-buffered saline (PBS) three times. Part of the tissue was fixed with 4% paraformaldehyde for 24 h and then embedded in paraffin. A second sample was used for the isolation and culture of gingival fibroblasts, and a third portion was applied to RNA/protein extraction and detection.

### Mutation screening and bioinformatics

Array-CGH was performed to determine whether chromosomal aberrations were present in the proband. To identify the pathogenic variant responsible for HGF, we performed whole-exome sequencing with genomic DNA for affected individuals II-1 and III-14 and the control II-5 in collaboration with Novogene-Beijing (China). We discovered two significant mutations located in *KIF3C* and *ZNF513* on chromosome 2 in two patients in the family. Short tandem repeat markers (D2S2168, D2S144, D2S2223, D2S174, D2S2247, D2S365, and D2S170), which flank *KIF3C* and *ZNF513*, were selected for haplotype analysis. Co-segregation analysis in 16 family members with Sanger sequencing was used to the inheritance of these two mutations.

To provide further insights into KIF3C and ZNF513 functionality, tertiary structures of the wild-type and mutant protein were predicted using ITASSER and Pymol. SIFT was used to predict the harmfulness of the mutations. ClustalX 2.0 and GeneDoc were used for multiple sequence alignment based on the NCBI database to analyze the conservation of affected amino acids across species. The two mutations were assessed with high-resolution melting (HRM) analysis in 300 unrelated control subjects with matched geographical ancestry.

### Plasmid construction, virus packaging, cell infection and transfection

Clustered Regularly Interspaced Short Palindromic Repeats (CRISPR)-Cas9, an adaptive immune system present in a variety of microorganisms, is a self-defense mechanism for bacteria to resist foreign pathogens. The CRISPR system, including non-coding RNA elements and enzymes, can recognize characteristic sequences and cleave foreign nucleic acids and can be used in eukaryotic cells for fast and effective gene editing. The CRISPR type II system is composed of the Cas9 protein and a single-guide RNA (sgRNA). By recognizing protospacer adjacent motifs (PAMs), sgRNAs pair complementarily with target DNA sequences. The Cas9 protein cleaves the sequence under the guidance of the sgRNA, resulting in a double-strand break, resulting in gene editing.^34,35^

According to the predicted score and specificity, we designed and synthesized three sgRNAs for *KIF3C* and *ZNF513* with a web tool (http://crispr.mit.edu). These were annealed to form double strands. The sgRNA annealing products were ligated with the expression vector (lentiCRISPR v52961), digested by *BsmB*I, and then CRISPR recombinant plasmids were obtained by competent cell transformation, monoclonal screening, and plasmid extraction. HEK-293T cells were inoculated on 10-cm petri dishes 1 day in advance, and plasmids transfection was carried out when the cells grew to 70%. LentiCRISPR v52961 recombinant plasmid, PSPAX2, and PMD2G in each petri dish were allocated in a ratio of 7.5 μg:7.5 μg:5 μg, while the ratio of PEI to total plasmid was 3 μg:1 μg. Plasmids and PEI were mixed with DMEM separately and then mixed together and added to the cells. After 8 h culturing, the supernatant was discarded, and 10 mL DMEM (with 10% FBS) was added. After 24 h and 48 h of culturing, 4 mL of complete medium was added. After 72 h of culturing, the supernatant of the cells was collected, and the virus suspension was obtained after ultracentrifugation for 1 h at 4 °C at 20 000 r·min^−1^, and re-suspension with DMEM.

Normal human gingival fibroblasts (NHGFs) were inoculated on 24-well plates in advance, and the cells grew to 70% confluence to be infected with the virus. The old medium was discarded, 500 μL complete medium containing 1/1 000 polybrence was added, and then 30 μL virus suspension was added. After 24 h of culturing, puromycin (final concentration 1–3 μg·mL^−1^) was added for screening. After continuous screening for 7 d, the infected cells were expanded, and PCR was employed to identify whether the gene was knocked out successfully. With flow cytometry, a single cell was selected from the cell suspension and inoculated in a 96-well plate, and the clone growth was observed after about 10 d of culture. After the cells were further expanded and cultured, DNA, RNA, and protein were extracted to verify whether *KIF3C* or *ZNF513* was knocked out completely.

The reference and mutant sequences of *KIF3C* and *ZNF513* were cloned into the lentivirus overexpression vector (pLenti-Bi-cistronic). The detailed steps of virus packaging and cell infection were the same as above.

In order to confirm the effect of two variants on NHGFs cells from another aspect, we cloned the sequences of *KIF3C* and *ZNF513* into expression vectors, pcDNA3.1flag and pEGFPN1, respectively. After being constructed, pcDNA3.1flag-(m)KIF3C and pEGFPN1-(m)ZNF513 overexpression plasmids were transiently transfect into NHGFs using Lipofectamine 2000 (Invitrogen) in Opti-MEM. 6–8 h after transfection, cells were cultured in fresh complete medium until they can be accessed for different purpose.

### Generation and genotyping of C57BL/6-*Kif3c*^R412H^ and C57BL/6-*Zfp513*^R250W^ knock-in mice

Human *ZNF513* corresponds to *Zfp513* in mice. A C57BL/6 mouse model with a point mutation (R412H) at the *Kif3c* locus and (R250W) at the *Zfp513* locus was generated by Saiye (Suzhou) Biological Technology Co., Ltd. with CRISPR/Cas9-mediated genome engineering. Briefly, the R412H (CGT to CAT) mutation in the NM_008445.2 transcript of *Kif3c* or the R250W (CGG to TGG) mutation in the NM_175311.4 transcript of *Zfp513* in the donor oligo was introduced into each exon by homology-directed repair. Cas9 mRNA, gRNA, generated by in vitro transcription, and donor oligos were co-injected into fertilized eggs for knock-in mouse production. F0 positive mice were selected and confirmed by PCR and DNA sequencing (Supplementary Fig. 7a, b).

The mouse *Kif3c* is located on mouse chromosome 12, while *Zfp513* is located on chromosome 5. Accordingly, six genotypes, *Kif3c*^R412H/R412H^/*Zfp513*^+/+^, *Kif3c*^+/+^/*Zfp513*^R250W/R250W^, *Kif3c*^+/R412H^/*Zfp513*^+/R250W^, *Kif3c*^R412H/R412H^/*Zfp513*^+/R250W^, *Kif3c*^+/R412H^/*Zfp513*^R250W/R250W^, and *Kif3c*^R412H/R412H^/*Zfp513*^R250W/R250W^, of mice were generated from the F1 *Kif3c*^+/R412H^/*Zfp513*^+/+^ and *Kif3c*^+/+^/*Zfp513*^+/R250W^ self-crossed inbred lines. Mouse DNA was isolated from tail biopsy samples and genotyped by PCR followed by sequence analysis (Supplementary Fig. 7c). All experiments involving mice were conducted in accordance with the Institutional Animal Care and Use Committee of Southern Medical University approved guidelines.

### Separation and processing of the murine mandible and peeling the maxillary gingiva

After the mice were sacrificed using approved guidance, we cut both sides of the oral cavity with a straight scissors, pulled down the murine mandible, excised the mandible including mandibular molar, and then trimmed away the irrelevant tissue. The separated mandible was fixed with 4% paraformaldehyde for 24 h and placed in decalcifying fluid for 3–4 weeks (replaced once every one week). The mouse mandibular first molar was cut on the coronal plane and then embedded in paraffin for serial sections and hematoxylin and eosin (HE) staining, as mentioned below.

The scissors were directed perpendicular to the plane of the palatal bone, and we incised tissues 1 mm behind the third molars and 2 mm behind the incisors. Then, we incised the vestibule until the posterior of both sides of the maxilla. We separated the maxilla from the rest of the skull, cut the middle suture, and trimmed the palatal tissue.^36^ The maxillary gingiva was stripped from the anterior border with tissue forceps for subsequent experiments. One part of the excised gingiva was used for the culture of gingival fibroblasts, and the second part was applied for RNA/protein extraction and detection.

### Primary human or murine gingival fibroblasts isolation, culture, and identification

In brief, rinsed human gingival tissues were trimmed into 1 mm × 2 mm squares with scissor in PBS (2% penicillin/streptomycin), and then these squares were digested in dispase II (2 mg·mL^−1^) at 4 °C for 16–18 h. The obtained subepithelial tissues were plated in DMEM (low glucose) with 20% FBS and 1% penicillin/streptomycin and cultivated for 5–7 days with the tissue adherent method. Identification of the gingival fibroblasts were confirmed by immunofluorescent staining against vimentin (positive) and cytokeratin (CK19, negative).

Separated mouse maxillary gingival tissues were cut into pieces in a tissue culture dish (35 × 10 mm^2^) with 1 mL PBS, 2% FBS, 2% penicillin/streptomycin, Collagenase Type IV (2 mg·mL^−1^), and DNAse Type I (1 mg·mL^−1^) and were incubated for 20 min at 37 °C at 200 r·min^−1^ in a shaker incubator.^36^ Next, 20 μL EDTA 0.5 M was added, and the tissues were incubated for another 10 min at 37 °C at 200 r·min^−1^. The centrifuged cell pellets were plated in 1640 (20% FBS, 1% penicillin/streptomycin) and cultivated for 4–6 days.

### Chromatin immunoprecipitation (ChIP)

ChIP is a method to study the interaction between proteins and DNA in living tissues or cells at the whole-genome level. According to the protocol of the EZ ChIP^TM^ Chromatin Immunoprecipitation Kit (Millipore), we collected 2 × 10^7^ NHGFs or patient gingival tumor fibroblasts, followed by formaldehyde cross-linking and ultrasonic fragmentation, pre-cleaning, and antibody incubation, immune complex precipitation and cleaning, purification, and recovery of the DNA samples. The parameters for ultrasonic breaking of DNA by the ultrasonic processor M220 (Covaris) were as follows: peak incident power: 65 W, sample volume: 60 μL, treatment time: 20 s on and off, repeats: 9, and total time: 6 min. Immunoprecipitation antibodies included a positive control (anti-RNA polymerase), negative control (rabbit IgG), and target antibody (ZNF513).

The obtained DNA products were used for qPCR to detect gene expression levels. After ChIP-qPCR, the value of %Input was calculated by the following specific formula (%Input = 2^((Cq(IN)−Log2(DF))−Cq(IP))^ × 100^37^; IN: Input; IP: Immunoprecipitated; DF: dilution factor), and agarose electrophoresis was employed to detect the qPCR products. Additionally, DNA products were used for PCR, whose primers were the same as the qPCR primers. After TA cloning with PCR products, positive clones were selected and identified as genes to be tested with Sanger sequencing.

### Histopathology and immunohistofluorescence staining

Human gingival biopsies were fixed with 4% paraformaldehyde for 24 h, then dehydrated in a series of alcohols, and finally embed in paraffin. Tissues were serially cut into 2-μm thick sections and stained with HE and Masson. In brief, paraffin gingiva sections were heated at 65 °C for 30 min and kept in xylene for deparaffinization. Next, the tissues were rehydrated in graded alcohols and stained for 10–15 min with hematoxylin, followed by differentiating in 1% hydrochloric acid alcohol for 5 s. 1% eosin was used for a 3 min staining, then the tissues were further dehydrated in graded alcohols and kept in xylene. Masson staining was performed with the trichromatic staining kit. After heating, deparaffinization, and rehydrating in xylene and graded alcohols, paraffin sections of the gingival tissues were stained for 5 min with Masson compound solution. In addition, 5 min phosphomolybdic acid, 5 min aniline blue staining, and 1 min differentiation liquid were used sequentially. After HE and Masson staining, a light microscope was used to carry out histopathological evaluation of human gingival tissues after mounting with neutral balsam.

After deparaffinization, rehydrating, and antigen retrieval, the paraffin sections were blocked with PBS containing 3% bovine serum albumin for 30 min. Polyclonal rabbit anti-KIF3C antibody (1:100, GeneTex) and polyclonal rabbit anti-ZNF513 antibody (1:100, Sigma) were used to incubate tissues at 4 °C overnight. Then, goat anti-rabbit IgG antibody (DyLight594, 1:200, GeneTex) was incubated for 1 h at room temperature away from light. Finally, the tissues were viewed using LSM 880 with Airyscan (Carl Zeiss, Jena, Germany) after mounting with DAPI medium.

### Cell proliferation

EdU is a thymidine analogue, and its alkyne group is rare in natural compounds. It can replace thymine (T) to infiltrate into synthetic DNA molecules during DNA replication. Based on the specific reaction of Apollo fluorescent dye with EdU, the replication activity of DNA can be detected directly and accurately. Stable gene overexpression cells, human or murine gingival fibroblasts, in the logarithmic growth phase were inoculated in a 96-well plate and cultured at 37 °C for 24 h. According to the instructions of the Cell-Light EdU Apollo567 In Vitro Kit (Ribobio), EdU labeling, cell fixation, Apollo staining, and DNA staining were performed sequentially. Finally, the positive cells were viewed under a fluorescence microscope and counted with ImageJ software.

Human gingival fibroblasts and stable gene overexpression cells were inoculated in 96-well plates at the same cell density. Transfected NHGFs were transferred at the same initial concentration and cultured in 96-well plates 24 h after transfection. After 12, 24, 36, 48 or 60 h of culturing, 100 μl of the culture medium containing 10 μL Cell Counting Kit-8 (CCK-8, UE) solution was added per well, and cells were incubated at 37 °C for 1.5 h. Living cells were evaluated with a microplate reader at 450 nm.

### Migration assay

The Transwell migration assay was used to quantify cellular migration. Human or murine gingival fibroblasts cultured to the third generation were prepared in 300 μL cell suspensions (2.5 × 10^5^ cells in serum-free medium). The migration chambers with a diameter of 6.5 mm and an aperture of 8 μm were placed in a 24-well plate, then 750 µL culture medium (with 10% FBS) was added to the lower chamber, followed by 300 µL cell suspension, and the plate was incubated at 37 °C for 24 h. After incubation, the medium was removed and the chamber was washed twice in PBS. The chamber was stained with 500 μL 0.1% crystal violet at room temperature for 20 min. The crystal violet was removed, and the chamber was washed twice by PBS, then we scraped off nonmigrated cells with cotton swabs, and finally observed the migrated cells under a light microscope. In addition, the stained chamber was placed in 400 μL 33% acetic acid solution shaking at room temperature for 10 min. 200 μL of the eluent was transferred into a 96-well plate, and the absorbance was measured with a microplate reader at 560 nm. The migration rate was calculated according to the cell density standard curve (Supplementary Fig. 4a).

### Primer design and detection of gene expression

The main primer sequences involved in this paper are shown in Table S2. The expression of genes, including *KIF3C*, *ZNF513*, *COL1A1*, *FN1*, *SOS1*, *KCNQ1*, *PIK3CA*, and *PIK3CB*, was detected by qPCR or western blotting.

### Supplementary information

Supplementary tables and figures
