# Supplementary material for: Double heterozygous pathogenic mutations in KIF3C and ZNF513 cause hereditary gingival fibromatosis
Source: Int J Oral Sci. 2023 Sep 26;15:46. doi: 10.1038/s41368-023-00244-1 (PMC10522663; doi:10.1038/s41368-023-00244-1)
Supplement: Supplementary file 1 — Supplementary tables and figures [file 41368_2023_244_MOESM1_ESM.docx]

**Supplementary Information**

**[Double heterozygous pathogenic mutations](https://pubmed.ncbi.nlm.nih.gov/34801929/) in *KIF3C* and *ZNF513* cause hereditary** **gingival fibromatosis**

Jianfan Chen*, Xueqing Xu*, Song Chen, Ting Lu, Yingchun Zheng, Zhongzhi Gan, Zongrui Shen, Shunfei Ma, Duocai Wang, Leyi Su, Fei He, Xuan Shang, Huiyong Xu, Dong Chen, Leitao Zhang^#^, Fu Xiong^#^

| Tissue | beta | r | p value |
| --- | --- | --- | --- |
| Brain - Anterior cingulate cortex (BA24) | 0.07025837 | 0.74383188 | 2.89E-32 |
| Brain - Caudate (basal ganglia) | 0.11826979 | 0.71804159 | 2.80E-40 |
| Brain - Cerebellar Hemisphere | 0.16946856 | 0.70458028 | 1.43E-33 |
| Brain - Putamen (basal ganglia) | 0.14442683 | 0.70236767 | 8.50E-32 |
| Kidney - Cortex | 5.38356614 | 0.69910527 | 9.99E-14 |
| Brain - Nucleus accumbens (basal ganglia) | 0.10532798 | 0.66730763 | 4.53E-33 |
| Brain - Cortex | 0.08461261 | 0.66294850 | 1.18E-33 |
| Brain - Amygdala | 0.10998634 | 0.65489736 | 5.59E-20 |
| Brain - Hippocampus | 0.09628800 | 0.63207017 | 2.27E-23 |
| Brain - Frontal Cortex (BA9) | 0.05742195 | 0.62997663 | 1.63E-24 |
| Brain - Cerebellum | 0.20246988 | 0.62655899 | 1.10E-27 |
| Brain - Hypothalamus | 0.09918646 | 0.60264908 | 2.34E-21 |

Table S1 Screening of tissues co-expressed by *KIF3C* and *ZNF513*

Beta: regression coefficient; r: correlation coefficient.

Table S2 Main primers

| Name | Sequence | Remark |
| --- | --- | --- |
| KIF3C-F/R | AGCTCAACCTCGTGGACCTG  CCTTGTACTTGGCCGCAAGC | KIF3C mutation |
| ZNF513-F/R | CCCAGTGGCCCGTGGTATAG  TACTCGAGCCACCTGAAGCG | ZNF513 mutation |
| pLentiK-F/R | CGCGGATCCAGCAGCAAGATGGCCA  CCGCTCGAGGGTTGTCACTCATGGT | KIF3C-pLenti plasmid |
| pLentiZ-F/R | AAGGAAAAAAGCGGCCGCATGCCCCGAAGGAAG  CTAGTCTAGATCAGGATGAGTCTGTG | ZNF513-pLenti plasmid |
| KsgRNA-F/R | caccgTGATGTAGACGCCAGTCTCG  aaacCGAGACTGGCGTCTACATCAc | KIF3C sgRNA |
| ZsgRNA-F/R | caccGTCGTGTCAGGTTGACGAGC  aaacGCTCGTCAACCTGACACGAC | ZNF513 sgRNA |
| K-HRM-F/R | CAGCTGGAGAAGAGGGGGAT  GCGGACACGGCCTTCTT | KIF3C HRM |
| Z-HRM-F/R | GTGTCTTACCTTCAGGTCGCC  TGCTGTACTCCACGACCAG | ZNF513 HRM |
| KIF3C-qF/R | TCCGGGACGAGGAGACTATG  TGGCGTAGAGCTTCTTGAGT | KIF3C qPCR |
| ZNF513-qF/R | ACTAGGCCAGGATCTGGAGTT  CTTCCGAGTCTCTCTCGAAGC | ZNF513 qPCR |
| Kchip-qF/R | CATTGAAACCCTGGAGCACG  ACCTTTGACGCCGTGTATGA | KIF3C ChIP qPCR |
| Schip-qF/R | CGGTGTTTCTAAGTGGTCTTTCC  TGGAGTTCATTTCTGGAGTGGG | SOS1 ChIP qPCR |
| Ksg-qF/R | TGGTCCGGGCCTCCTATTT  TTGATGTAGACGCCAGTCTCG | KIF3C sgRNA qPCR |
| Zsg-qF/R | CCTTCGTGTCCCACTACTCG  TCGTGTCAGGTTGACGAGC | ZNF513 sgRNA qPCR |
| SOS1-qF/R | TTATCCAAGTTACTCTGCCCCA  TTCTGGTCGTCTTCGTGGAG | SOS1 qPCR |
| COL1A1-qF/R | CTGAGCCAGCAGATCGAGAA  CACATCTTGAGGTCACGGCA | COL1A1 qPCR |
| FN1-qF/R | CCGCCGAATGTAGGACAAGA  AGGGTTCTTCATCAGTGCCA | FN1 qPCR |
| KCNQ1-qF/R | CCCGCGCGTCTCCATCTA  AAACGAAGCATTTCCAGCCG | KCNQ1 qPCR |
| PIK3CA-qF/R | GGTTTGGCCTGCTTTTGGAG  CCATTGCCTCGACTTGCCTA | PIK3CA qPCR |
| PIK3CB-qF/R | TTTGGGAAAATAACAACCCTTTCC  ACCAGCCCTGACATGAACTTT | PIK3CB qPCR |
| M-Kif3c-F/R | TCCGAGTAGGCAAGCTCAAC  TTCTCCTCCTCCAGCCGTT | Kif3c mutation |
| M-Zfp513-F/R | GCACCCATACTGGCGAGAAG  GTTCGGATCCAGTTCCACACA | Zfp513 mutation |
| M-Kif3c-qF/R | CCGTCCACAACTAAAGTGCG  AGGAACAGAGGTCATGGATGC | Kif3c qPCR |
| M-Zfp513-qF/R | CTGTGGCTTTCGATGCTGTG  TCAGCGCATCTTCTGATCGT | Zfp513 qPCR |
| M-Sos1-qF/R | GAGCCGGACATCAAGAGGTT  TTAGGGTGCCGGGGTTCTAT | Sos1 qPCR |
| M-Col1a1-qF/R | CGCATGGCCAAGAAGACATC  AGCATACCTCGGGTTTCCAC | Col1a1 qPCR |
| M-Fn1-qF/R | AGCAAATCGTGCAGCCTCAA  TGCCTAGGTAGGTCCGTTCC | Fn1 qPCR |

**
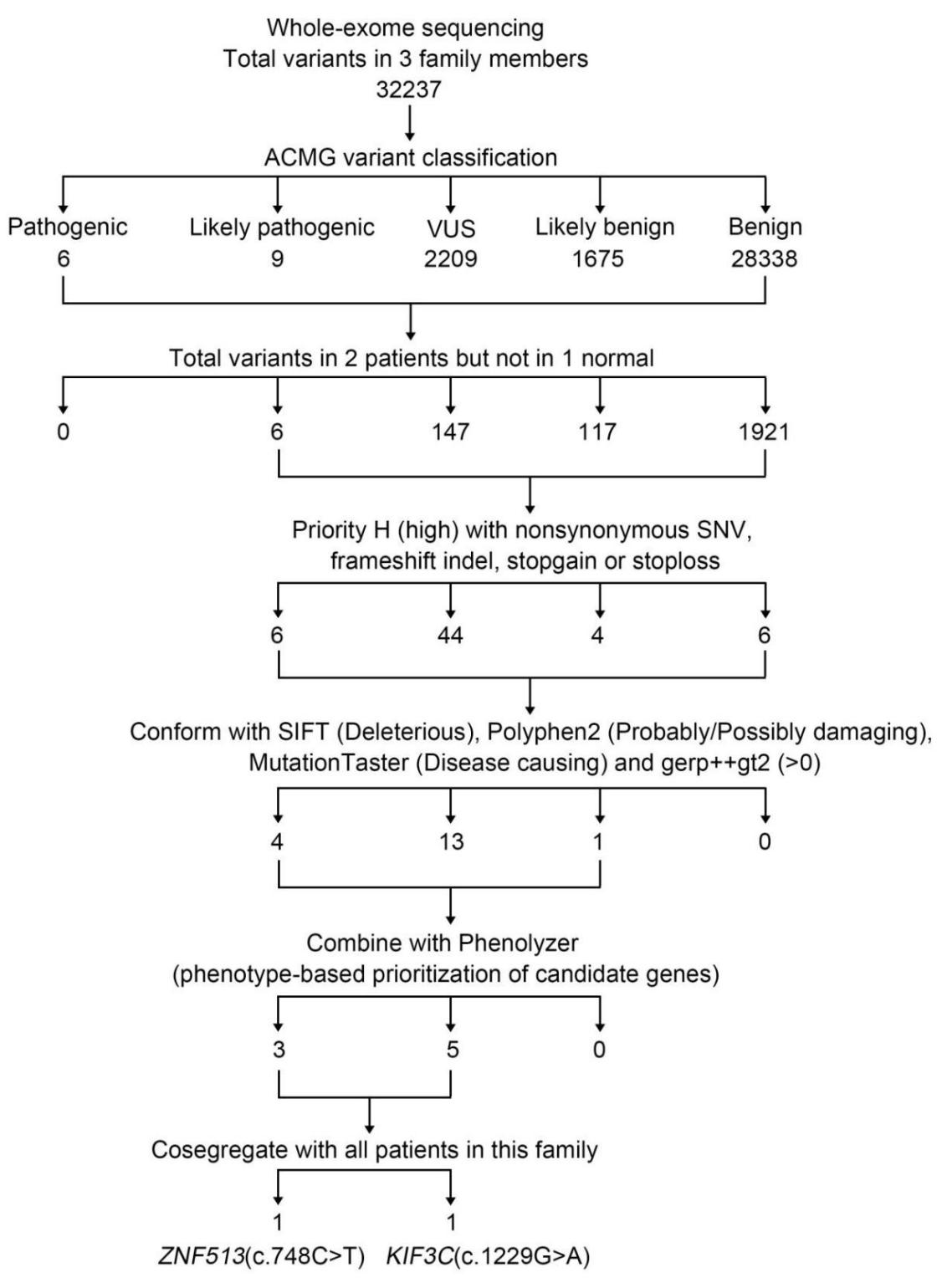
**

**Figure S1.** Screening candidate genes from whole-exome sequencing.


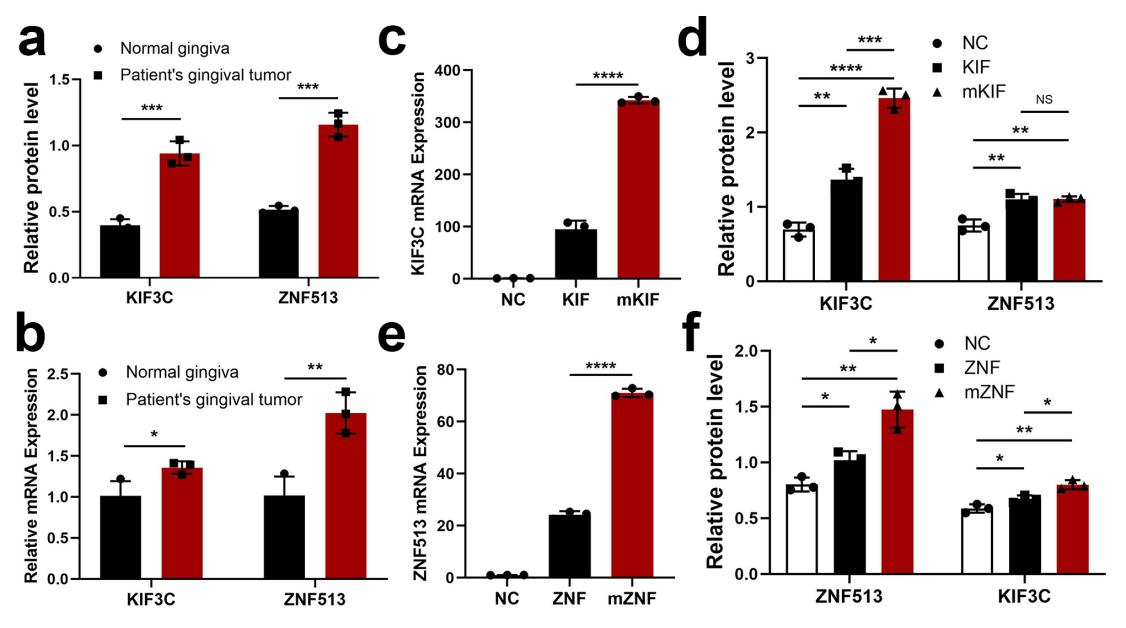


**Figure S2.** Effects of the two mutations on *KIF3C* or *ZNF513* expression in human gingival biopsies, primary gingival fibroblasts and stable lentivirus overexpressing cell lines. (**a**) By immunoblot analysis, the protein levels of KIF3C and ZNF513 in the patient’s gingival tumor were significantly increased. (**b**) The mRNA expression levels of *KIF3C* and *ZNF513* in normal primary human gingival fibroblasts and the patient’s gingival tumor fibroblasts. (**c** and **d**) The mRNA expression levels and the protein levels of the two genes in HGF-KIF3C and HGF-mKIF3C cell lines. (**e** and **f**) The expression levels of the two genes in HGF-ZNF513 and HGF-mZNF513 cell lines.


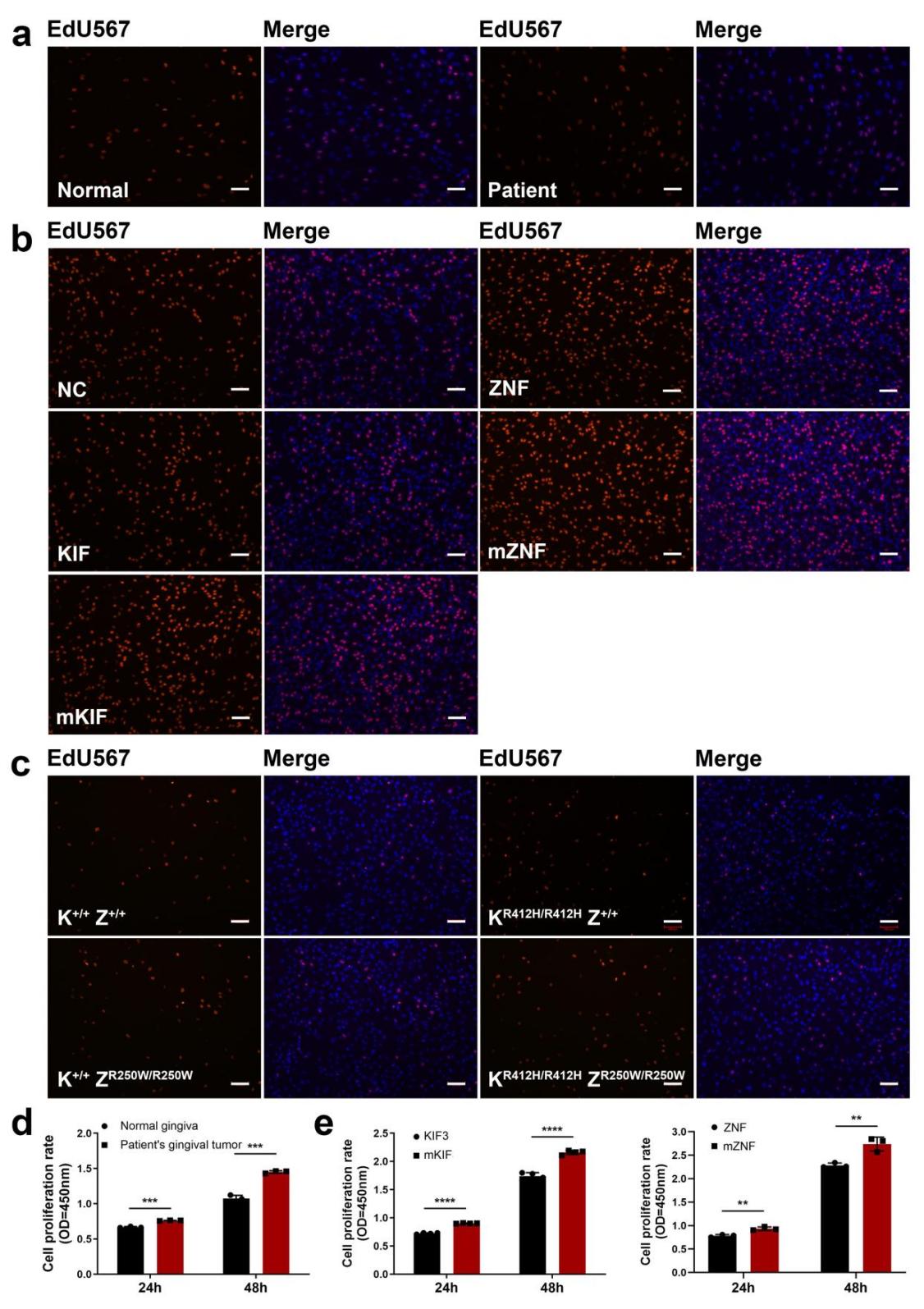


**Figure S3.** Using EdU and CCK-8 to detect cell proliferation. (**a** and **d**) Normal primary gingival fibroblasts and patient’s gingival tumor fibroblasts (EdU, CCK-8). (**b** and **e**) Stable lentivirus overexpressing *KIF3C* and *ZNF513* cell lines (EdU, CCK-8). (**c**) Primary fibroblasts from the maxillary gingiva of male mice (EdU). Object lens up to 10 magnifications. Scale bars indicate 100 μm.


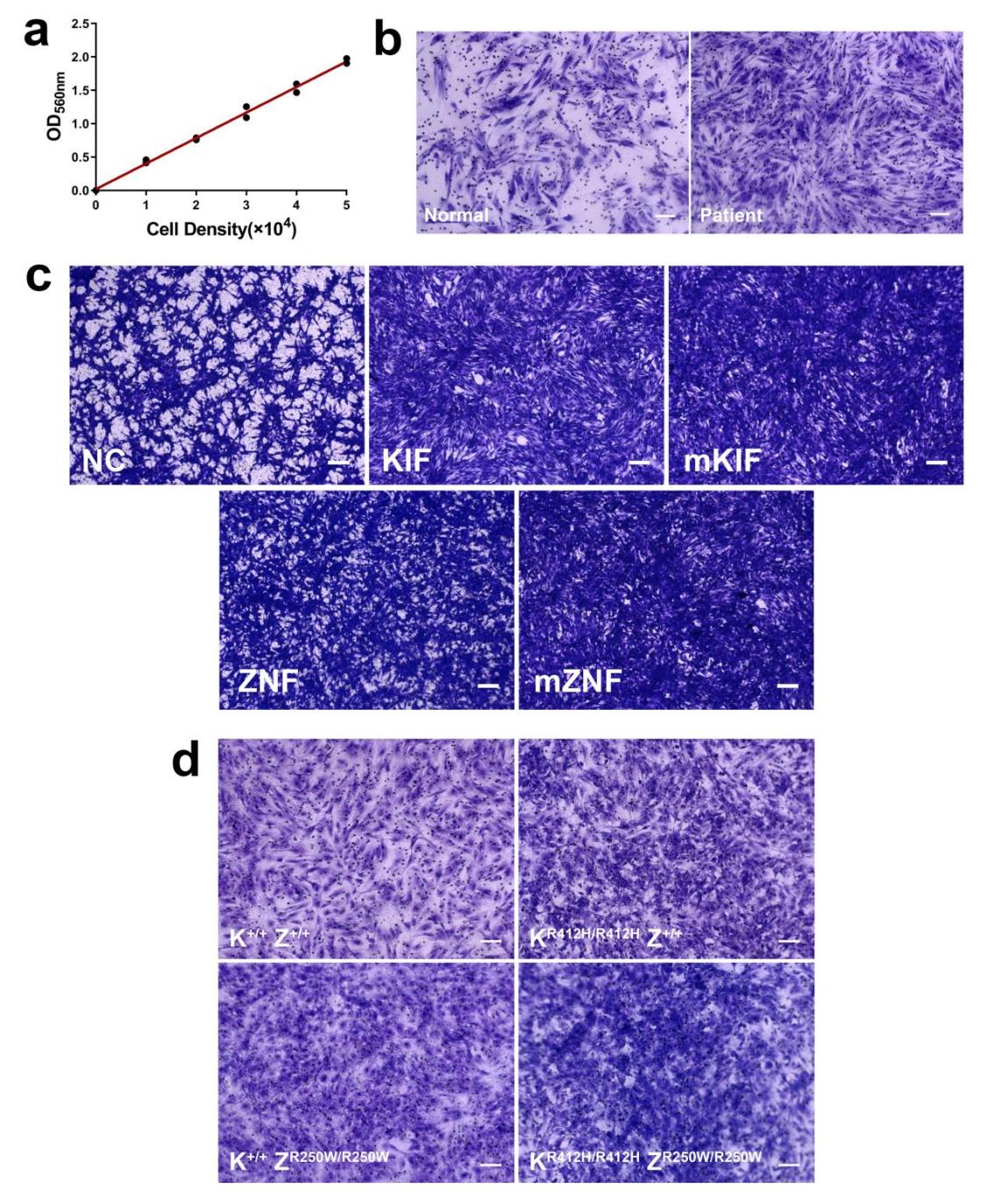


**Figure S4.** Cell migration. (**a**) The standard curve of the Transwell assay using NHGFs. Y = 0.3824x + 0.01983 (r^2^ = 0.9942). (**b**) Normal primary gingival fibroblasts and the patient’s gingival tumor fibroblasts. (**c**) Stable lentivirus overexpressing *KIF3C* and *ZNF513* cell lines. (**d**) Primary fibroblasts from the maxillary gingiva of male mice. Object lens up to 10 magnifications. Scale bars indicate 100 μm.


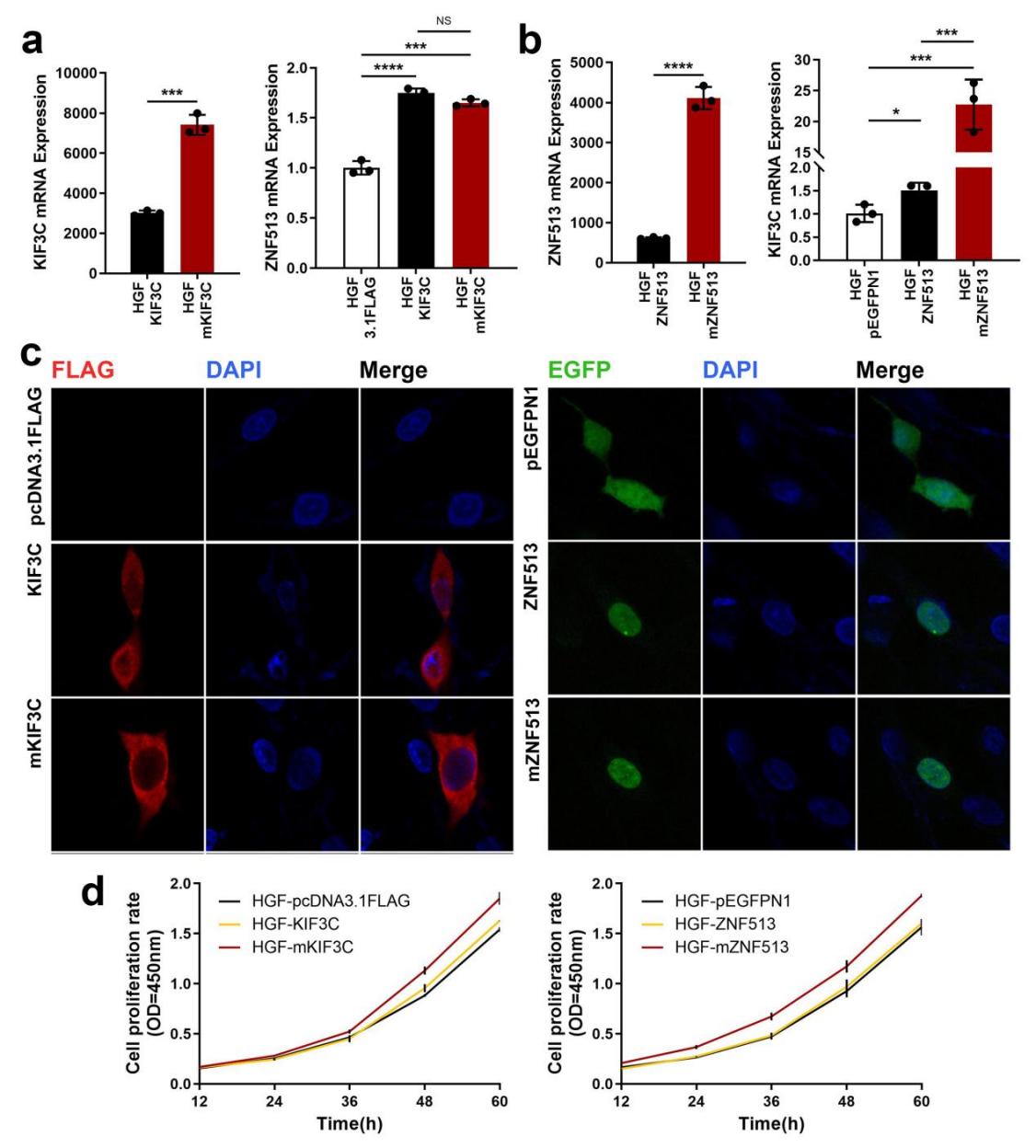


**Figure S5.** Transient transfection of HGF with *KIF3C* and *ZNF513*. (**a**) Overexpression of *KIF3C* and detection of *KIF3C* and *ZNF513* expression. (**b**) Overexpression of *ZNF513* and detection of *ZNF513* and *KIF3C* expression. (**c**) Protein localization of KIF3C and ZNF513 was performed after transient transfection of overexpression plasmids. (**d**) After transfection, cell proliferation was detected by CCK-8.


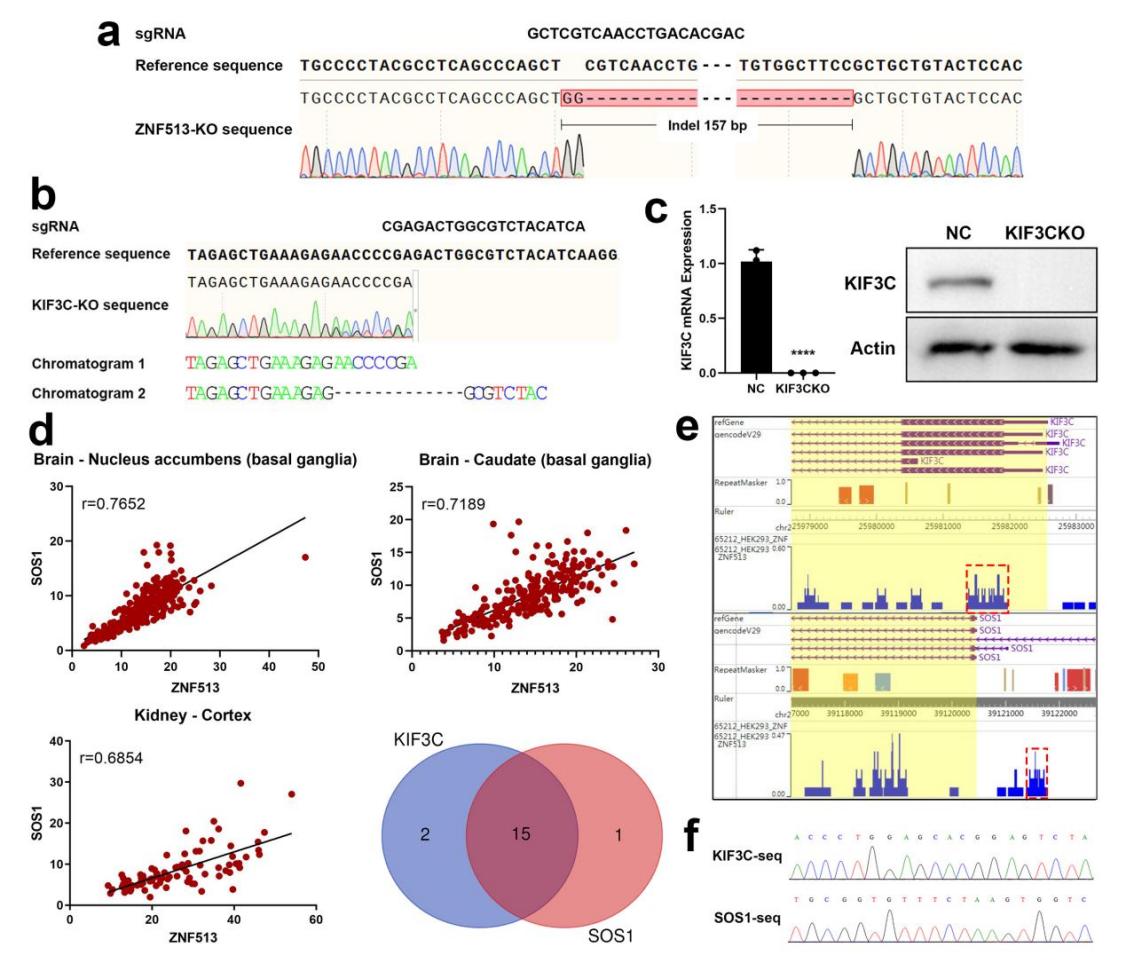


**Figure S6.** (**a**) Sanger sequencing of the *ZNF513* knockout HGF cell line. (**b**) Sanger sequencing of the *KIF3C* knockout HGF cell line. (**c**) *KIF3C* knockout HGF cell line was constructed by CRISPR/Cas9, and mRNA and protein were detected. (**d**) GTEx database analysis showed that there was co-expression and positive correlation between *ZNF513* and *SOS1* in many tissues. Venn diagram showing that the intersection of | r | > 0.5 was screened in the tissues co-expressed by *ZNF513*, *KIF3C*, and *SOS1*. (**e**) The ChIP data of ZNF513 in HEK293 cells in the Cistrome DB database showed that there were multiple binding site-rich regions in *KIF3C* exon 1 and the *SOS1* promoter. The red dotted frames are the region selected in this study. (**f**) ChIP-seq.


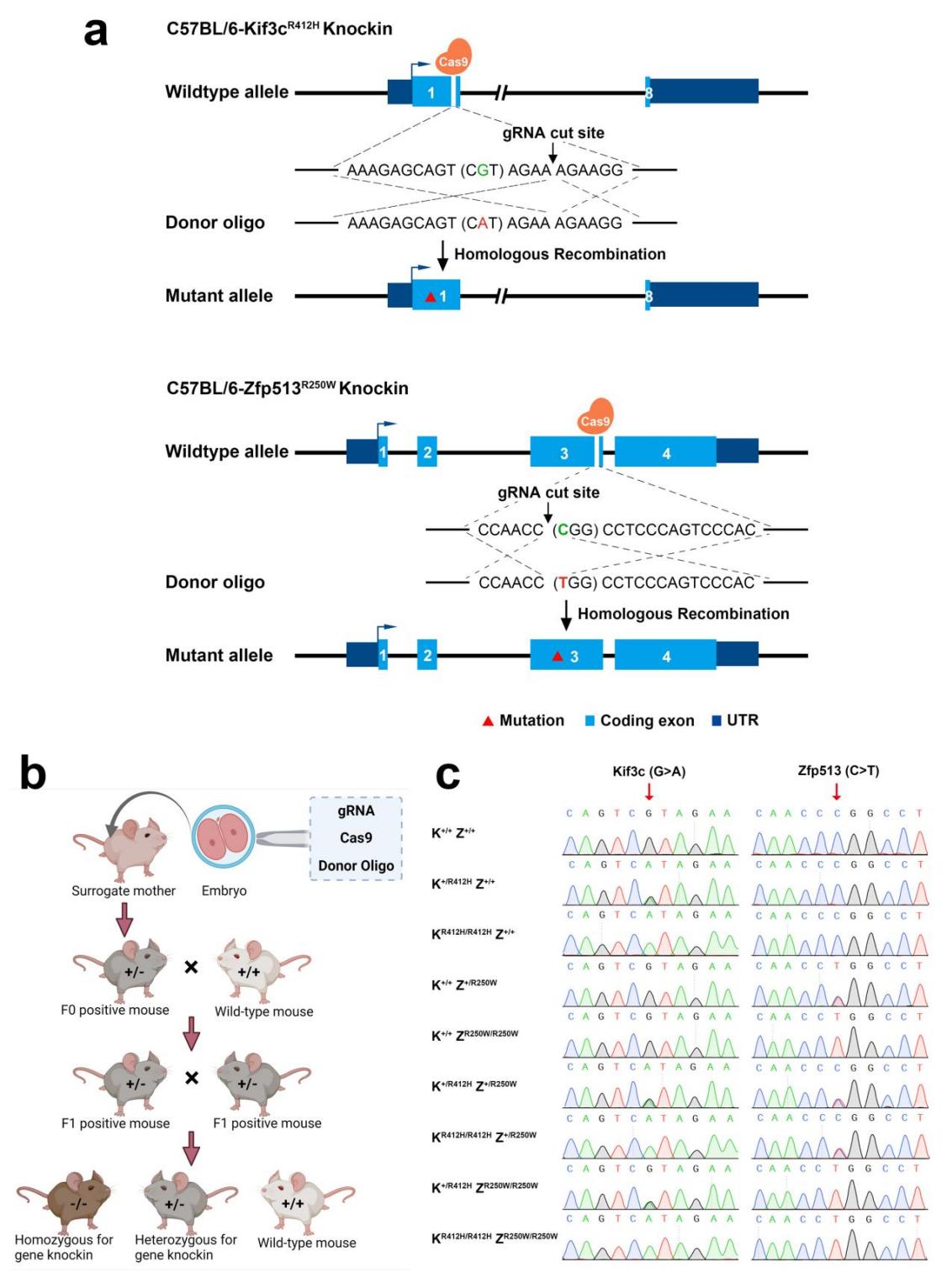


**Figure S7.** Knock-in mice. (**a** and **b**) Establishment of CRISPR/Cas9-mediated point mutation model of C57BL/6 mice. (**c**) Sanger sequencing to identify the mouse genotypes.
